# Evaluation of Different Gases and Gas Combinations for On-Farm Euthanasia of Pre-Weaned Pigs

**DOI:** 10.3390/ani8030040

**Published:** 2018-03-16

**Authors:** Nikki Kells, Ngaio Beausoleil, Craig Johnson, Mhairi Sutherland

**Affiliations:** 1 School of Veterinary Science, Massey University, Palmerston North 4442, New Zealand; n.j.kells@massey.ac.nz (N.K.); n.j.beausoleil@massey.ac.nz (N.B.); c.b.johnson@massey.ac.nz (C.J.); 2 AgResearch Ltd., Ruakura Research Centre, Hamilton 3240, New Zealand

**Keywords:** behaviour, electroencephalogram, euthanasia, pigs, welfare

## Abstract

**Simple Summary:**

There are times on a swine farm when pigs become ill or injured and must be euthanised. Blunt force trauma to the head is currently the most commonly employed method for on-farm euthanasia of pre-weaned piglets. When performed correctly, loss of consciousness is rapid, but the potential for delivery of sub-lethal blows, along with aesthetic unacceptability to many operators, has led to the need for alternative methods to be developed. The practice of using carbon dioxide (CO_2_) to euthanise piglets during the pre-weaning period is becoming more common on-farm in the United States; however, animals may display behavioural and/or physiological signs of stress or aversion in response to CO_2_ inhalation. Inducing anoxia using argon (Ar) gas may cause less aversion or stress and thus be preferable to using CO_2_. Therefore, the aim of this research was to evaluate the effects of 100% CO_2_, 100% Ar or CO_2_ and Ar combined (60% Ar/40% CO_2_) on piglet welfare during euthanasia. The results from this research suggest that using CO_2_, Ar or a 60% Ar/40% CO_2_ mixture causes stress to piglets prior to loss of consciousness and hence alternative methods of euthanasia need to be evaluated.

**Abstract:**

The aim of this research was to evaluate the welfare of pre-weaned piglets euthanised using three different gas treatments: 100% carbon dioxide (CO_2_), 100% argon (Ar) or a mixture of 60% Ar/40% carbon dioxide (Ar/CO_2_). Two studies (n = 5 piglets/treatment/study) were conducted: (1) behavioural and physiological data were collected from conscious piglets during exposure to test gases via immersion in a pre-filled chamber and (2) electrophysiological data were collected from lightly anaesthetised, intubated and mechanically ventilated piglets exposed to the same test gases. Based on the duration of escape attempts and laboured breathing, piglets exposed to 100% CO_2_ experienced more stress than piglets exposed to 100% Ar prior to loss of consciousness, but there appeared to be no advantage of mixing Ar with CO_2_ on indices of animal welfare. However, spectral analysis of the electroencephalogram revealed no changes consistent with nociception during exposure to any of the three gas treatments. Based on the behavioural response to gas exposure, all gases tested caused signs of stress prior to piglets losing consciousness and hence alternative methods of euthanasia need to be evaluated.

## 1. Introduction

In the course of swine husbandry, there are occasions when piglets become ill or injured and farm workers must decide if euthanasia is necessary, and if so, which method is most appropriate. The American Veterinary Medical Association (AVMA) guidelines on euthanasia [1] endorse several methods of euthanasia as being acceptable for pre-weaned piglets. These include anaesthetic overdose (e.g., using barbiturates), carbon dioxide (CO_2_) inhalation and blunt force trauma performed manually or using a purpose built non-penetrating captive bolt [1]. Of these, anaesthetics are regulated substances that require appropriately qualified personnel to obtain and administer. In practical terms, this means employing the services of a veterinarian, which may not always be practical and may be cost prohibitive when large numbers of animals are involved. Whilst euthanizing piglets using a purpose built non-penetrating captive bolt, manual blunt force trauma or CO_2_ do not pose these problems, there are nonetheless still potential welfare and worker safety issues involved with their use. The AVMA Guidelines on Euthanasia, developed to provide guidance on how to prevent and/or relieve suffering of animals that are to be euthanized, stipulates that methods should induce rapid loss of consciousness preceding death and should minimise any distress and anxiety experienced prior to loss of consciousness [1]. In addition, the psychological well-being of personnel performing the procedure should be considered. 

Blunt force trauma performed manually [2,3] or using a purpose built non-penetrating captive bolt [4,5,6] can induce rapid loss of consciousness in neonatal pigs up to 10.9 kg (approximately 50 days of age). However, correct placement and sufficient force are necessary to ensure rapid loss of consciousness when performing this procedure [4,6,7]. Even when performed accurately, these methods may be aesthetically unappealing to those carrying out or witnessing the procedure [2,8]. In contrast, gaseous methods of euthanasia may be perceived more positively by some farm workers [9]. Moreover, gas euthanasia has the additional advantage that the pig does not need to be restrained. However, specialised equipment in the form of a chamber and gas delivery system are needed for this method of euthanasia. Overall, there are advantages and disadvantages to using either blunt force trauma or gas to euthanise pre-weaned piglets. Both methods involve potential risks to worker safety and/or animal welfare if equipment is not maintained or the procedures are not performed correctly. Nonetheless, there is a need to evaluate different methods of euthanasia for pre-weaned piglets so as to be able provide producers with options that suit their particular production system.

Several gases are currently used to euthanise, stun or kill animals, including CO_2_ and the inert gases, argon (Ar) and nitrogen [10,11,12,13]. Gaseous methods are routinely used for laboratory rodents, with CO_2_ being the most commonly used agent because it is heavier than air and thus containable, cheap to obtain and relatively safe for human operators [14,15,16,17]. The use of CO_2_ is also common for stunning pigs in slaughter plants in the US and Europe [13]. High concentrations of CO_2_ cause central nervous system depression leading to loss of consciousness and subsequent death. However, prior to this there is the potential for animals to experience severe breathlessness due to hypercapnia [18,19,20], as well as pain due to the formation of carbonic acid in the nasal and respiratory mucosa [21,22]. Moreover, behavioural and/or physiological signs of distress in response to CO_2_ inhalation have been reported in rats [15], broiler chickens [10,23] and pigs [24,25]. 

It has been suggested that oxygen deprivation is a more humane method of euthanasia than CO_2_ inhalation [26]. This can be accomplished with the use of inert gases, such as Ar, that displace oxygen in air. Oxygen deprivation results in hypoxia which leads to loss of consciousness and subsequent death as neurons become starved of oxygen [1]. Because Ar is an odourless, non-irritant gas, it is thought that loss of consciousness and death via Ar-induced anoxia may occur with little or no aversion or distress. Finishing pigs readily entered a chamber containing 90% Ar for a food reward but avoided entering a chamber containing 90% CO_2_ [27]. Furthermore, exposure to 2% oxygen in Ar induced minimal respiratory stress compared to 90% CO_2_ in pigs [24]. Therefore, Ar maybe preferable to 100% CO_2_ as a method of euthanasia for pigs based on indicators of animal welfare. The other advantage of using Ar is that it is a non-flammable and non-explosive gas, making it a practical choice from a work safety stand point. Pigs exposed to 2% oxygen in Ar took twice as long to lose consciousness (latency to loss of posture) as pigs exposed to 90% CO_2_ [27], potentially reducing the practicality of using Ar alone as a method of on-farm euthanasia for piglets. However, mixing Ar with CO_2_ could potentially reduce the time to loss of posture in pigs and cause less stress than exposing pigs to 90% CO_2_ [24]. A 50:50 mixture of Ar and CO_2_ did not improve the welfare of neonatal piglets during gas exposure compared with 100% CO_2_ [12]. However, in an approach-avoidance test, piglets appeared to find a gas mixture of 60% Ar/30% CO_2_ less aversive than 90% CO_2_ [11]. Furthermore, humans report pain when exposed to CO_2_ concentrations of 50% or greater [28], and nociceptors in the rat nasal mucosa begin to respond to CO_2_ concentrations between 37% and 50% [29]. Therefore, a concentration of 40% CO_2_ was selected based on the desire to avoid/reduce potential nociceptor stimulation, and thus pain perception, without markedly increasing the latency to loss of consciousness for practical purposes. The objectives of this study were to evaluate the welfare of pre-weaned piglets euthanised using 100% CO_2_, 100% argon (Ar) or a mixture of 60% Ar/40% carbon dioxide (Ar/CO_2_) using (1) behavioural and physiological and (2) electrophysiological indicators of stress. 

## 2. Materials and Methods 

All research was undertaken with prior approval from the Massey University Animal Ethics Committee (protocol 10/22 [study 1] and 10/58 [study 2]).

Data collection was undertaken in two parts. In study one, behavioural and physiological data were collected from conscious piglets during exposure to test gas mixtures via immersion in a purpose-built euthanasia chamber pre-filled with the test gas. In study two, electrophysiological data were collected from lightly anaesthetised, intubated and mechanically ventilated piglets exposed to the same test gas mixtures via an endotracheal tube. Electrophysiological data were collected in the first study; however, electroencephalograph (EEG) data were incomplete due to contamination of the recordings caused by voluntary (escape behaviour) and involuntary (convulsions) skeletal muscle activity which necessitated the need to repeat the study. In the second study, piglets were lightly anaesthetised and given a neuromuscular blocking agent (atracurium) to inhibit skeletal muscle activity and prevent subsequent contamination of EEG recordings. In both study one and two, healthy and viable pigs were used as we did not want to confound health status, such as respiratory disease, with the effects of gas euthanasia [30].

All piglets were commercial white-line (Landrace × Large white) entire males, sourced from a local commercial pig farm. At approximately 17 days of age, pre-weaned piglets were collected from the farm in the morning of the test day. Following transportation in a closed vehicle to the university, the animals were group housed in a 28 °C temperature-controlled ventilated room, with deep straw bedding over non-slip concrete flooring. Piglets had *ad libitum* access to water and commercial creep feed until the time of testing. Immediately prior to testing each piglet was transported individually to the laboratory in an enclosed pet carry-cage.

### 2.1. Study 1: Physiological and Behavioural Response to Gas Euthansia

Fifteen male piglets, approximately 17 days of age (range 14 to 20 days) and weighing 4.2 ± 1.14 kg (mean ± SD), were sourced from seven different litters and were randomly assigned to receive one of three test gas treatments: 100% carbon dioxide (CO_2_, n = 5), 100% (Ar, n = 5) or a mixture of 60% Ar/40% carbon dioxide (Ar/CO_2_, n = 5). 

A purpose-built plastic 60 L chamber (558 length × 390 height × 327 width mm) was used to administer the gas treatments. The lid and one side of the chamber contained a clear perspex viewing window to provide unobstructed viewing and video recording of piglet behaviour throughout the procedure. Gas inlet and outlet hoses were located at one end of the chamber. The gas outlet hose was attached at a higher level than the inlet hose to facilitate displacement of ‘lighter’ air. A sealed port was situated in the lid of the chamber to allow instrument cables to be connected to the chart recorder.

On each occasion the chamber was pre-filled with the appropriate test gas, sourced from cylinders of compressed CO_2_, Ar and medical grade air (Air Liquide New Zealand Ltd., Auckland, New Zealand) connected to the chamber. Gas was introduced at a rate of 10 L/min (20% of the chamber volume) for 10 min prior to the animal being introduced and was maintained throughout the test period at this flow rate. 

Prior to placement in the chamber, each piglet was instrumented as follows: A custom-made adjustable fabric belt was placed around the piglet’s abdomen to monitor respiration rate. The belt was fitted with a saline-filled pressure cuff connected to a force transducer, which in turn connected to a chart recorder to allow continuous recording of chest excursions related to respiration. Subcutaneous 27-gauge stainless steel needle electrodes (Viasys Healthcare, Surrey, UK) were positioned to record EEG and electrocardiograph (ECG) activity. A five-electrode montage was used to record EEG from both the left and right cerebral hemispheres, with inverting electrodes placed parallel to the midline over the left and right frontal bone zygomatic processes, non-inverting electrodes over the left and right mastoid processes and a ground electrode placed caudal to the occipital process. ECG was recorded using a base-apex configuration. A small amount of contact adhesive was used to prevent potential displacement of the electrodes during data recording. Both EEG and ECG signals were fed via breakout boxes to separate amplifiers (Iso-Dam isolated biological amplifier, World Precision Instruments Inc., Sarasota, FL, USA). The signals were amplified with a gain of 1000 and a band-pass of 1.0–500 Hz and digitised at a rate of 1 kHz (Powerlab 4/20, ADInstruments Ltd., Colorado Springs, CO, USA). The digitised signals were recorded on an Apple Macintosh personal computer for analysis off-line at the conclusion of the experiment. During instrumentation, piglets remained relatively calm.

Following instrumentation, the lid of the pre-filled chamber was quickly lifted, the piglet was placed on its feet in the centre of the chamber and the lid quickly secured. During gas exposure, behaviour was continuously recorded until death using two digital video recorders (Sony DCR-SR20, Sony Corporation, Tokyo, Japan) positioned outside the chamber to record dorsal and lateral views. Death was confirmed by isoelectric EEG and ECG in conjunction with respiratory arrest. In case of the piglet injuring themselves or equipment failure, pentobarbital was on hand as an alternative method of euthanasia. 

#### 2.1.1. Physiological Measures

A 5 mL blood sample was collected into an EDTA-vacutainer by jugular venepuncture immediately prior to the piglet being placed in the chamber and once death was confirmed. Plasma was immediately separated by centrifugation and stored at −80 °C for later analysis of cortisol and epinephrine concentrations. Plasma samples were tested in duplicate according to the manufacturer’s instructions, using commercially available cortisol (R&D Systems Parameter Cortisol Assay KGE008, R&D Systems, Minneapolis, MN, USA) and epinephrine (Alpco AP17-EPIHU-e01 Epinephrine assay, Alpco Diagnostics, Salem, NH, USA) enzyme immunoassay test kits.

Raw EEG, ECG and respiration data were recorded continuously for 10 min prior (baseline) to and during gas exposure. EEG was visually inspected and classified into one of three categories: active, transitional or isoelectric [31]. EEG was classified as active when the amplitude was the same as that observed prior to placement in the chamber (baseline EEG); transitional EEG was defined as having an amplitude less than 50% of baseline EEG; isoelectric EEG was classified as a stable trace consisting of background noise with an amplitude <1/8 that of baseline. Raw ECG was also examined to determine when cessation of cardiac electrical activity (irreversible cardiac arrest) occurred.

#### 2.1.2. Behavioural Measures

Video recordings from both camera views were examined and the behaviour of each animal was scored according to the ethogram defined in Table 1. One trained observer, blinded to the treatments, scored the behaviour of each piglet from the time when the lid was secured after the piglet was placed in the chamber until the time that death was confirmed.

### 2.2. Study 2: Electrophysiological Response to Gas Euthansia

Fifteen male piglets, approximately 17 days of age (range 14 to 22 days) and weighing 4.6 ± 0.89 kg (mean ± SD), were obtained from nine different litters and were randomly assigned to receive one of three test gases: 100% carbon dioxide (CO_2_, n = 5), 100% argon (Ar, n = 5), or a mixture of 60% argon/40% carbon dioxide (Ar/CO_2_, n = 5).

In preparation for testing, a piglet was placed into a custom-built perspex chamber and anaesthesia was induced with 5% halothane vaporised in air (4 L/min). Once adequate anaesthesia was achieved (indicated by lateral recumbence and absence of withdrawal reflex to a toe pinch), endotracheal intubation was carried out using a 3.5–5.0 mm cuffed endotracheal (ET) tube. After confirmation of successful intubation by capnometry, the ET tube was connected to a breathing circuit and anaesthesia was maintained with halothane in air. Piglets were mechanically ventilated (18–20 cm H_2_O maximum inspiratory pressure) with end-tidal CO_2_ maintained in the normocapnic range (40–50 mmHg). Halothane delivery was reduced to achieve an end-tidal tension of 1.2 ± 0.5%, which was maintained throughout. Stainless steel needle electrodes were positioned as described in study one to monitor EEG and ECG activity. After electrode placement, a 22-gauge venous cannula was inserted in an auricular vein for the administration of a neuromuscular blocking agent (atracurium) to inhibit skeletal muscle activity and prevent subsequent contamination of electrophysiological recordings. Atracurium (Tracrium^®^; GlaxoSmithKline, Melbourne, Australia) was given at a rate of 1 mg/kg. A digital rectal thermometer was used to continuously monitor body temperature. The animal was placed on top of a warm-water heating blanket device (T pump; Gaymar Industries Inc., New York, NY, USA) to help maintain body temperature throughout. A Doppler plethysmograph was positioned over the radial artery of the foreleg to monitor blood flow, and to identify the point at which blood flow ceased due to cardiac failure. When end-tidal halothane was stable at 1.2 ± 0.5% and end-tidal CO_2_ was stable in the normocapnic range, baseline EEG/ECG was recorded for 10 min.

Test gases were administered following the 10 min baseline period. Treatment gas was administered from a separate anaesthetic system, through a second vaporiser (calibrated to the first to ensure the same halothane concentration) to allow a virtually instantaneous switch from room air to test gas, mimicking study one in which piglets were placed in a pre-filled chamber.

Continuous monitoring of heart rate, pupil reactivity and lacrimation was carried out to ensure maintenance of anaesthesia throughout the procedure. End tidal halothane and CO_2_ tensions, body temperature and blood flow were monitored throughout.

EEG and ECG data were recorded continuously until death (as determined by isoelectric EEG and the cessation of cardiac contractile activity), at which time gas exposure ceased. EEG was analysed off-line at the conclusion of the experiment.

#### EEG and ECG Analysis

Raw EEG data were visually inspected and classified into active, transitional or isoelectric waveforms, as described for Study one. EEG data were band pass filtered between 0.1 and 100 Hz during recording. Frequency spectra were generated for sequential non-overlapping 1 s epochs of recorded data. Briefly, data were low-pass filtered at 30 Hz and Fast Fourier Transformation applied to each epoch, yielding sequential frequency spectra with a resolution of 1 Hz. From these, the summary variables median frequency (F50), 95% spectral edge frequency (F95) and total power (PTOT) were derived. For each animal, baseline values for F50, F95 and PTOT were obtained by calculating the mean value for each variable over the final 5 min of baseline immediately preceding test gas exposure. Mean values for consecutive 5 s blocks from the start of gas exposure were then calculated, and transformed to a percentage of baseline mean.

ECG data were band-pass filtered between 1.0 and 500 Hz during recording. Raw ECG recordings were used to generate heart rate for sequential 1 s epochs using the ratemeter function in LabChart 8.1 (ADInstruments Ltd., Dunedin, New Zealand).

### 2.3. Statistical Analysis

All statistical analyses were carried out in SAS 9.1.3 (SAS Institute Inc., Cary, NC, USA). Plots of standardized residuals versus predicted values were evaluated to test the assumption of normally distributed within-group errors, centred at zero with constant variance. Where the residuals were found to approximate a normal distribution, parametric analyses were carried out, whilst non-parametric tests were used to analyse data with non-normally distributed residuals. All values are reported as non-transformed mean ± standard error of the mean (SEM).

For study one, all data were subjected to non-parametric one-way analysis of variance (Kruskal-Wallis test), followed by the post-hoc Bonferroni’s test for multiple comparisons when a significant main effect was found. For study two, latency to the appearance of transitional and isoelectric EEG, along with maximum heart rate and time to cessation of blood flow, was subjected to non-parametric analysis of variance (Kruskal-Wallis test), with Bonferroni’s post-hoc tests for multiple comparisons carried out where a significant treatment effect was identified.

Fourier-transformed EEG data and heart rate data from study two were analysed using the mixed procedure in SAS with age, weight and treatment as fixed effects, piglet as a random effect and time as a repeated measure. Where a significant treatment effect was identified, *p*-values were manually corrected for multiple comparisons by dividing by the number of comparisons of interest. Differences were considered significant at *p* < 0.05.

## 3. Results

### 3.1. Study 1: Physiological and Behavioural Response to Gas Euthansia

#### 3.1.1. Physiological Measures

Plasma cortisol concentrations increased in response to treatment, irrespective of gas treatment (change in cortisol concentrations (ng/mL): CO_2_: 7.6 ± 21.86, Ar: 18.7 ± 21.86, Ar/CO_2_: 24.0 ± 21.86; *p* = 0.811). Plasma epinephrine concentrations increased in response to treatment and there was a tendency for a smaller increase following exposure to Ar/CO_2_ than Ar (change in epinephrine concentrations (ng/mL): CO_2_: 24.7 ± 2.30, Ar: 26.1 ± 2.30, Ar/CO_2_: 16.9 ± 2.30; *p* = 0.075).

EEG data were unavailable for four individuals, due to the displacement of recording electrodes as a result of vigorous escape behaviour during the experiment. Of the remaining 11 datasets, EEG data were incomplete due to contamination of the recordings caused by voluntary (escape behaviour) and involuntary (convulsions) skeletal muscle activity. The precise time to appearance of a transitional EEG waveform could not be determined for any individual. The time to appearance of isoelectric EEG could only be precisely determined for three individuals; therefore no further data analysis was conducted. In addition, the lack of clean ECG or respiration rate data following transfer to the chamber precluded further analysis of these variables.

#### 3.1.2. Behavioural Measures

The mean time to onset of specific behaviours identified in the ethogram (Table 1), along with number and duration of events (where applicable), are shown in Table 2. With the exception of latency to onset and duration of convulsions, onset of gasping and respiratory arrest, values shown refer to those events observed in the period prior to the onset of loss of posture, which is the first indication of loss of consciousness [24].

Escape attempts were observed in all individuals. The latency to onset (*p =* 0.007), frequency (*p =* 0.01) and duration (*p* < 0.001) of escape attempts occurred sooner, more often and for a longer duration in piglets exposed to 100% CO_2_ compared to those exposed to 100% Ar. Latency, frequency and duration of escape attempts did not differ between Ar/CO_2_ and CO_2_ treated piglets.

There was no effect of gas treatment on frequency or duration of squealing; however, piglets exposed to the 100% Ar treatment performed more (*p* < 0.05) grunts, for a longer (*p* < 0.05) duration, than piglets exposed to either CO_2_ or Ar/CO_2_ treatments.

All animals in the CO_2_ and Ar/CO_2_ treatments showed evidence of laboured breathing following transfer to the test chamber, whereas only two of the five piglets in the Ar treatment exhibited laboured breathing. Laboured breathing occurred sooner and was performed for longer before loss of posture in CO_2_ (*p* < 0.001 and *p* = 0.001, respectively) and Ar/CO_2_ (*p* < 0.001 and *p* = 0.005, respectively) treated piglets compared with Ar treated piglets. In addition, laboured breathing was rated as being more (*p* < 0.05) intense when piglets were exposed to 100% CO_2_ than 100% Ar, but did not differ between Ar and Ar/CO_2_ treatments. Prior to placement in the chamber, all piglets exhibited normal quiet breathing.

Loss of posture occurred sooner in CO_2_ and Ar/CO_2_ (*p* = 0.045 and *p* = 0.004, respectively) than Ar treated piglets. Loss of posture immediately preceded the onset of convulsions in all animals and convulsions were observed in all individuals. No piglets regained postural control after cessation of initial convulsions.

The latency to onset of convulsions was shorter in the CO_2_ (*p* = 0.045) and Ar/CO_2_ (*p* = 0.004) than the Ar treatment. The number and total duration of convulsions was greater in the Ar than the CO_2_ (*p* = 0.001 and *p* < 0.001, respectively) or Ar/CO_2_ (*p* = 0.003 and *p* = 0.002, respectively) treatments.

Gasping was observed in all individuals, following apparent loss of consciousness. In 10 of 15 piglets (67%), gasping commenced prior to the cessation of convulsions. Gasping was observed sooner in piglets exposed to the CO_2_ or Ar/CO_2_ treatments compared with Ar (*p* = 0.034 and *p* = 0.025, respectively).

Respiratory arrest occurred sooner in piglets exposed to the CO_2_ treatment compared with those exposed to Ar (*p* < 0.001) or Ar/CO_2_ (*p* = 0.006).

Head shaking, coughing or sneezing were not observed in any individuals. Only three of 15 piglets urinated or defecated during exposure to the gas, with this occurring after loss of posture on all occasions. Therefore, these behaviours are not presented in Table 2.

### 3.2. Study 2: Electrophysiological Response to Gas Euthansia

#### EEG Response

The appearance of a transitional EEG waveform occurred sooner in the CO_2_ than in the Ar treatment and intermediate in the Ar/CO_2_ treatment (*p* = 0.033; Table 3). Isoelectric EEG was observed sooner in piglets exposed to the CO_2_ treatment than those exposed to Ar (*p* = 0.033) and tended to occur sooner in CO_2_ treated than Ar/CO_2_ treated piglets (*p* = 0.059; Table 3).

**Figure 1:**
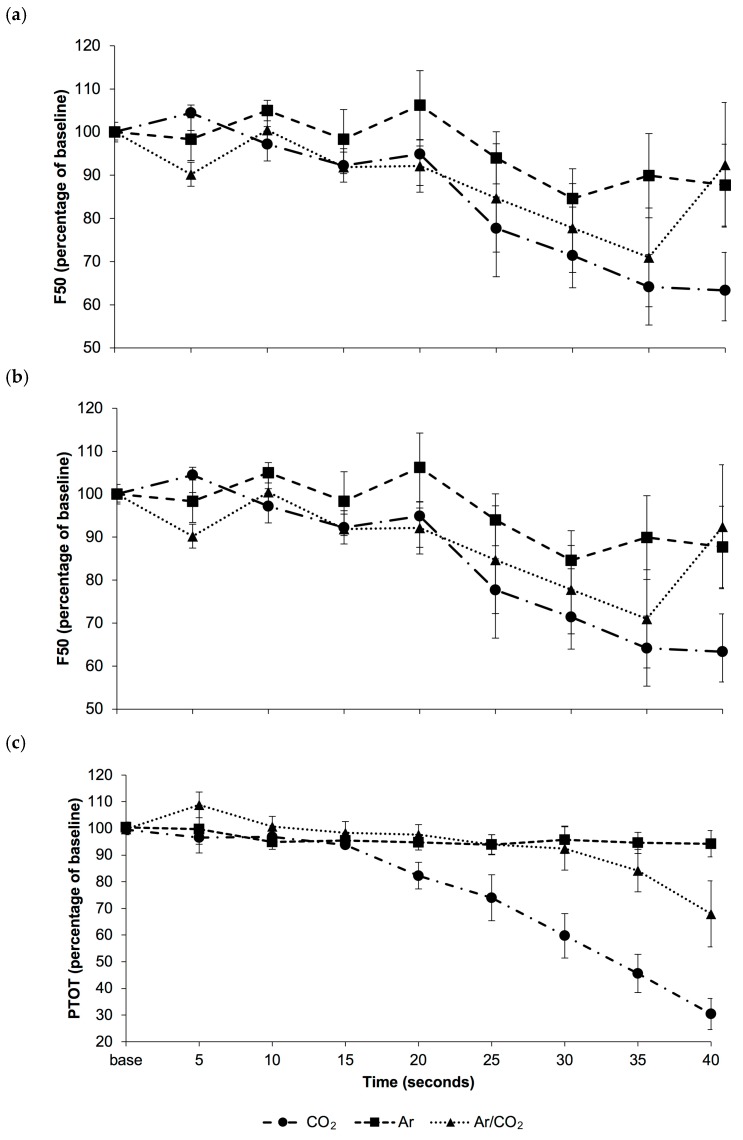
Mean (± SEM) changes in (**a**) median frequency (F50), (**b**) spectral edge frequency (F95) and (**c**) total power (PTOT) of the EEG over time in piglets subjected to gas euthanasia (n = 5/treatment) with 100% carbon dioxide (CO_2_), 100% argon (Ar) or a mixture of 40% carbon dioxide/60% argon (Ar/CO_2_).

Changes in F50, F95 and PTOT over the first 40 s of exposure to treatment gases, relative to baseline, are shown in Figure 1. Data beyond 40 s exposure time are not presented, as EEG was transitional in 5/5 piglets in the CO_2_ group and 1/5 piglets in the Ar/CO_2_ group beyond this point.

There was an effect of time on the change in F50 of the EEG (*p* < 0.001), whereby F50 was lower than baseline from 25–40 s after gas exposure (Figure 1a). There was an effect of time on the change in F95 of the EEG (*p* = 0.014), with mean F95 tending to be lower than baseline after 35 s of gas exposure (*p* = 0.071; Figure 1b). There were effects of treatment (*p* < 0.001) and time (*p* < 0.001), along with a treatment × time interaction (*p* < 0.001), on the change in PTOT of the piglet EEG. Total power decreased more rapidly following exposure to CO_2_ than to either Ar or Ar/CO_2_ (Figure 1c) and was significantly lower than baseline from 30 s. Total power in the CO_2_ treatment was lower than the Ar treatment after 25 (*p* = 0.033), 30, 35 and 40 s (*p* < 0.001), and lower in the Ar/CO_2_ treatment after 25 (*p* = 0.032), 30 (*p* < 0.001), 35 and 40 s (*p* < 0.001). After 40 s of gas exposure, PTOT was also lower in the Ar/CO_2_ than the Ar treatment (*p* = 0.003). 

## 4. Discussion

The purpose of this research was to evaluate the welfare of pre-weaned piglets euthanised using 100% CO_2_, 100% Ar or a mixture of 60% Ar/40% CO_2_. Of the gases evaluated, it appears that 100% CO_2_, whilst inducing the most rapid loss of consciousness and death, also results in the greatest negative impact on welfare prior to loss of consciousness. In contrast, 100% Ar appeared to have the least impact on welfare of the gas treatments. Escape behaviours, such as retreating, raising the forelegs against the sides of the chamber, and running or charging at the walls or lid of the chamber are considered evidence of stress or aversion in pigs exposed to hypoxic or hypercapnic atmospheres [13,24,32]. Escape behaviours were observed in response to all three gas treatments, but occurred sooner and for a longer duration in piglets exposed to 100% CO_2_ than in those exposed to 100% Ar. Laboured breathing, potentially indicative of unpleasant sensations of breathlessness, also occurred sooner and for a longer duration in piglets exposed to 100% CO_2_ than in those exposed to 100% Ar, as well as being rated as more intense. Breathlessness in response to hypercapnia is described as being highly unpleasant in humans [18,19], therefore laboured breathing was deemed indicative of reduced welfare in piglets in the present study. Our results are consistent with a previous study which reported that pigs exposed to various concentrations of CO_2_ experienced respiratory distress sooner, and for a greater duration and intensity, than pigs exposed to Ar with 2% residual oxygen [24]. A more marked response in terms of escape behaviours and laboured breathing, suggests that piglets experience more stress or aversion prior to loss of consciousness when exposed to 100% CO_2_ than 100% Ar. Moreover, duration and latency to perform escape behaviours and laboured breathing was similar for piglets exposed to 100% CO_2_ or 60% Ar/40% CO_2_, suggesting that there is no advantage of mixing these gases from an animal welfare perspective. Similarly, Sadler et al. [12] concluded that there was no advantage of euthanizing neonatal piglets by placing them into a chamber prefilled with a 50% Ar/50% CO_2_ mixture compared with 100% CO_2_ based on parameters associated with animal welfare. However, piglets appeared to find a 60% Ar/30% CO_2_ mixture less aversive than 90% CO_2_ in an approach-avoidance test, though the Ar mixture still adversely affected the piglets and caused them to leave the test chamber [11]. Therefore, it would be worth evaluating the welfare implications of euthanizing pre-weaned pigs using gas mixtures containing lower concentrations of CO_2_ or other inert gases.

Interestingly, vocalisations were only performed by piglets exposed to Ar (Ar and Ar/CO_2_ treatments). In previous studies evaluating the suitability of hypoxic and hypercapnic gas atmospheres for stunning pigs, squealing prior to loss of consciousness has been interpreted as a sign of stress or aversion [27,33,34]. Furthermore, squealing is also associated with stress in response to handling and painful husbandry procedures in piglets [35]. We speculate that the marked hyperventilation observed in piglets exposed to 100% CO_2_ may have affected their ability to vocalise. Moreover, grunting prior to loss of posture was only observed in the 100% Ar group. Grunting generally occurred soon after the piglet was placed in the chamber, while it appeared to investigate the novel environment. No grunting was recorded during the performance of escape behaviours. Given that grunting appeared to be associated with exploratory behaviour, it may indicate that Ar is less aversive to piglets, at least initially, than the CO_2_ or Ar/CO_2_ treatments tested in this study.

Activation of the sympathetic nervous system and hypothalamic-pituitary-adrenal axis have previously been shown to occur in response to stress in piglets, such as painful husbandry procedures [36,37,38]. Plasma cortisol and epinephrine concentrations were elevated in all piglets following euthanasia in the present study, irrespective of gas treatment, suggesting that all treatments elicited a stress response. Similarly, cortisol concentrations were elevated in piglets gradually exposed to 100% CO_2_ at a flow rate of 20% chamber volume per minutes [25,39] and in piglets placed in a chamber pre-filled with 100% CO_2_ [25]. In addition, nor-epinephrine concentrations were elevated in piglets euthanised with 100% CO_2_ [39]. However, pre-treatment concentrations of cortisol and epinephrine in the present study would not be reflective of actual baseline values as piglets had already experienced several stressors prior to testing, including separation from the sow, transport, handling, instrumentation and blood sampling. In addition, it is not possible to differentiate between the stress experienced by the piglets prior to loss of consciousness (e.g., exposure to the gas, a novel environment, isolation) or the physiological changes that occurred after loss of consciousness. Hence, in the present study, cortisol and epinephrine were likely of limited value as indicators of welfare when evaluating methods of euthanasia. 

It should be acknowledged that piglets in the present study were exposed to a number of potential stressors prior to being placed in the euthanasia chamber (e.g., transport, instrumentation for EEG recording etc.) and prior to loss of conscious whist in the chamber (e.g., isolation, exposure to a novel environment etc.). Exposure to these stressors could potentially have affected the piglets’ subsequent responses to gas exposure in study one. However, given that piglets in each of the gas treatments were exposed to the same stressors, these are unlikely to account for the behavioural differences observed among the gas treatments tested. It would be of interest to differentiate between the stress response caused by gas inhalation alone and that associated with the other stressors experienced by the piglets as part of the euthanasia process, however this was beyond the scope of this study. The measurements of EEG does allow the assessment of nociception without the confounding effects of external stressors, hence assessing welfare using behaviour in conscious animals and electrophysiological changes in unconscious animals may provide a good model for assessing the welfare implications of different gaseous methods of euthanasia in piglets.

Loss of consciousness may be inferred behaviourally, based on loss of posture [24] or by examining changes in the amplitude or power of the EEG [31,40]. Loss of posture coincided with the onset of convulsions in all piglets, limiting the use of this measure as an indicator of loss of awareness in the present study. On the other hand, analysis of EEG in study two revealed that cortical suppression, which leads to loss of conscious awareness in non-anaesthetised animals, occurred sooner in the CO_2_ than the Ar treated piglets, based on the development of transitional and isoelectric EEG waveforms. In conscious animals, transitional EEG is thought to be indicative of loss of awareness, whilst isoelectric EEG is deemed incompatible with consciousness [40]. The appearance of these waveforms in lightly anaesthetised pigs provides an indication of the relative rates of cortical depression induced by the test gases. Based on this, the duration of potential negative experience may be shortest when piglets are euthanised using 100% CO_2_. However, despite the likely faster onset of unconsciousness with CO_2_, conscious piglets exposed to 100% CO_2_ still performed escape behaviours and laboured breathing for longer prior to loss of consciousness than piglets exposed to 100% Ar.

Spectral analysis of EEG revealed no clear evidence of nociception prior to onset of transitional EEG waveform for any of the gas treatments tested in the present study. Given that transitional EEG likely indicates loss of awareness, analysis of the EEG beyond this point was not carried out. The typical pattern of mammalian cortical response to noxious stimulation is a transient increase in the F50 along with a transient decrease in the PTOT of the EEG in the period immediately following application of the noxious stimulus [41,42,43,44]. For example, noxious stimulation in the form of a tail clamp applied to rats, or electrical stimulus applied to the hind limb of dogs, elicited changes in F50 and PTOT of the EEG within 10 s of stimulus application [44,45]. Previous studies have shown that nociceptors in the nasal mucosa of humans and rats respond to CO_2_ concentrations of 30 to 40% [21,28,29], and that inhalation of 40 to 55% CO_2_ reportedly causes pain in the eyes, nose and throat of humans, through stimulation of nociceptors in the mucosa of the corneal, nasal and upper respiratory regions [21,22]. Therefore the absence of a nociceptive response, particularly to 100% CO_2_, in this study was unexpected. Whilst the absence of a clear nociceptive response in the EEG of piglets exposed to either 40% or 100% CO_2_ in this study may suggest that CO_2_ does not induce a nociceptive response in young pigs, it is more likely that methodological limitations prevented the detection of any such response. The method employed to measure EEG in the present study required the animals to be intubated, preventing exposure of the corneal, nasal or upper respiratory tract mucosa to the test gas, which may have affected our results. Further study is required to rule out a nociceptive response to different gasses in pre-weaned piglets.

## 5. Conclusions

This study has identified that 100% Ar is preferable in welfare terms to 100% CO_2_ or a mixture of 60% Ar/40% CO_2_ for on-farm euthanasia of pre-weaned piglets. Furthermore, there appeared to be no advantage of using a mixture of 60% Ar/40% CO_2_ to euthanise piglets. However, piglets euthanised with 100% Ar still experienced some degree of stress or aversion in the period prior to loss of consciousness, as evidenced by performance of escape attempts and a brief period of laboured breathing before loss of posture and convulsions. Since an ‘ideal’ euthanasia method should induce no (or at least minimal) pain, stress or anxiety prior to loss of consciousness, there is still a need to continue to evaluate alternative on-farm methods of euthanasia that meet these criteria. 

## Figures and Tables

**Table 1:** Ethogram of piglet behaviour recorded during exposure to various gases.

Behaviour	Definition	Variables Recorded
Convulsions	Involuntary contraction of skeletal muscles, may be tonic ^1^, clonic ^1^ or both; includes paddling ^1^ motion of the limbs	Latency to onset, frequency and total duration of bouts ^2^
Escape attempt	Backing away or purposeful and vigorous butting of head, snout or shoulder into the chamber lid or walls or raising of forelegs against chamber walls	Latency to onset, frequency, duration
Gasping	Low frequency (≤4/min), very deep breathing through a wide open mouth, accompanied by large abdominal movements and stretching of the neck	Latency to onset
Head shaking	Vigorous, rapid, purposeful movement of head from side to side (at least 2 consecutive movements)	Frequency
Laboured breathing	Increase in rate and/or depth of ventilation compared with baseline (prior to transfer to chamber)	Latency to onset, total duration, intensity ^3^
Loss of coordination	Loss of balance, stumbling, or diminished muscle control	Latency to onset
Loss of posture	Animal collapses into a recumbent position, with no evidence of postural control, and does not regain posture, or show further evidence of awareness	Latency to onset
Respiratory arrest	Permanent cessation of respiratory movement (minimum of 60 s without a breath)	Latency to onset
Sneezing/Coughing	Animal sneezed or coughed	Frequency
Elimination	Evacuation of the bladder or bowels	Frequency
Vocalisation	Piglet emits an audible squeal or grunt	Type (squeal or grunt), number and total duration of bouts ^2^

^1^ Tonic: prolonged generalised contraction, clonic: alternating contraction/relaxation in quick succession, paddling: involuntary walking/running/galloping motion of the limbs. ^2^ A bout was defined as either a single discreet event, or a period of continuous events with <1 s pause. A pause of >1 s signalled the end of a bout. ^3^ A four-point scale was used to grade the intensity of laboured breathing, where 0 = closed mouth, with no evidence of a change in rate or depth (no visible abdominal movements); 1 = closed mouth, visible abdominal movement indicating increased depth, rate >1 breath/s; 2 = open mouth, evidence of increased rate and/or depth; 3 = gasping (as defined above) [24].

**Table 2:** Treatment least squares means (± SEM) for piglet behaviour during euthanasia with 100% carbon dioxide (CO_2_, n = 5), 100% argon (Ar, n = 5) or a mixture of 40% CO_2_/60% argon (Ar/CO_2_, n = 5).

Variable	CO_2_	Ar	Ar/CO_2_	*p*-Value
Latency to first escape attempt (s)	3.9 ± 2.2 ^a^ (min 0, max 6.5)	19 ± 2.9 ^b^ (min 11.5, max 29)	8.0 ± 2.2 ^a^ (min 4, max 12)	0.007
Number of escape attempts ^1^	2.4 ± 0.35 ^a^ (min 1, max 4)	0.6 ± 0.35 ^b^ (min 0, max 1)	1.2 ± 0.35 ^ab^ (min 1, max 2)	0.010
Duration of escape attempts (s)	7.2 ± 0.64 ^a^ (min 6, max 10)	1.3 ± 0.64 ^b^ (min 0, max 3.5)	2.8 ± 0.64 ^a^ (min 2, max 4)	<0.001
Number of squeals ^1^	0 ± 0.0	0.6 ± 2.4 (min 0, max 1)	0.6 ± 2.4 (min 0, max 1)	0.088
Duration of squeals (s) ^1^	0 ± 0.0	1.9 ± 0.61 (min 0, max 4.5)	0.4 ± 0.61 (min 0, max 1.0)	0.114
Number of grunts ^1^	0 ± 0.0 ^a^	1.4 ± 0.39 ^b^ (min 0 , max 4)	0 ± 0.0 ^a^	0.04
Duration of grunts (s) ^1^	0 ± 0.0 ^a^	5.4 ± 2.8 ^b^ (min 0, max 10)	0 ± 0.0 ^a^	0.003
Latency to laboured breathing (s)	5.8 ± 1.1 ^a^ (min 4, max 7)	21.2 ± 1.1 ^b^ (min 16, max 27)	4.6 ± 1.1 ^a^ (min 4, max 6)	<0.001
Duration of laboured breathing ^1^	8.6 ± 1.3 ^a^ (min 6, max 11)	3.0 ± 1.3 ^b^ (min 0, max 9)	6.8 ± 1.3 ^ab^ (min 4, max 9)	0.028
Intensity of laboured breathing (s) ^1^	2.0 ± 0.18 ^a^ (min 2, max 2)	0.4 ± 0.18 ^b^ (min 0, max 1)	1.8 ± 0.18 ^a^ (min 1, max 2)	0.003
Latency to loss of coordination (s)	9.5 ± 2.4 (min 5, max 13)	16.5 ± 2.4 (min 10, max 30)	8.5 ± 2.4 (min 5, max 13.5)	0.066
Latency to loss of posture ^1^	14 .4 ± 1.6 ^a^ (min 13, max 16)	20.8 ± 1.6 ^b^ (min 16, max 30)	11.4 ± 1.6 ^a^ (min 10, max 14)	0.004
Latency to first convulsion (s) ^2^	14.4 ± 1.6 ^a^ (min 13, max 16)	20.8 ± 1.6 ^b^ (min 16, max 30)	11.4 ± 1.6 ^a^ (min 10, max 14)	0.004
Number of convulsive bouts ^2^	4.6 ± 1.1 ^a^ (min 2, max 7)	12.2 ± 1.1 ^b^ (min 8, max 15)	5.4 ±1.1 ^a^ (min 2, max 9)	<0.001
Duration of convulsions (s) ^2^	20.3 ± 5.3 ^a^ (min 12, max 32)	71.6 ± 5.3 ^b^ (min 55, max 93)	36.7 ± 5.3 ^a^ (min 28, max 50)	<0.001
Latency to gasping (s) ^2^	33.2 ± 4.9 ^a^ (min 20, max 46)	84.2 ± 20 ^b^ (min 48, max 151)	30.2 ± 5.7 ^a^ (min 19, max 51)	0.013
Respiratory arrest (s) ^2^	113 ± 15.3 ^a^ (min 98, max 134)	331 ± 15.3 ^b^ (min 293, max 415)	225 ± 15.3 ^c^ (min 181, max 252)	<0.001

^1^ Measures recorded prior to loss of posture. ^2^ Measures recorded after loss of posture. ^a,b,c^ Means in the same row with different superscripts are significantly different at *p* < 0.05.

**Table 3:** Mean (± SEM) latency (seconds) to the appearance of transitional and isoelectric electroencephalogram (EEG) in piglets during gas euthanasia with 100% carbon dioxide (CO_2_, n = 5), 100% argon (Ar, n = 5) or a mixture of 40% CO_2_/60% argon (Ar/CO_2_, n = 5).

EEG Classification	CO_2_	Ar	Ar/CO_2_	*p*-Value
Transitional EEG	32.9 ± 2.92 ^a^	61.1 ± 4.53 ^b^	48.9 ± 7.73 ^ab^	0.03
Isoelectric EEG	46.2 ± 2.10 ^a^	69.0 ± 5.02 ^b^	67.1 ± 6.30 ^ab^	0.011

^a,b^ Means in the same row with different superscripts are significantly different at *p* < 0.05.

## References

[1] American Veterinary Medical Association AVMA Guidelines for the Euthanasia of Animals: 2013 edition. https://www.avma.org/KB/Policies/Documents/euthanasia.pdf.

[2] Chevillion P., Mircovich C., Dubroca S., Fleho J.Y. (2004). Comparison of different pig euthanasia methods available to farmers. Proc. Int. Soc. Anim. Hyg..

[3] Widowski T.M., Elgie R.H., Lawlis P. (2008). Assessing the effectiveness of a non-penetrating captive bolt for euthanasia of newborn piglets. Proc. Allen D. Leman Swine Conf..

[4] Casey-Trott T.M., Millman S.T., Turner P.V., Nykamp S.G., Widowski T.M. (2013). Effectiveness of a nonpentrating captive bolt for euthanasia of piglets less than 3 d of age. J. Anim. Sci..

[5] Casey-Trott T.M., Millman S.T., Turner P.V., Nykamp S.G., Lawlis P.C., Widowski T.M. (2014). Effectiveness of a non-penetrating captive bolt for euthanasia of 3–9 kg pigs. J. Anim. Sci..

[6] Grist A., Murrell J.C., McKinstry J.L., Knowles T.G., Wotton S.B. (2017). Humane euthanasia of neonates I: Validation of the effectiveness of the Zephyr EXL non-penetrating captive-bolt euthanasia system on neonate piglets up to 10.9 kg live-weight. Anim. Welf..

[7] Finnie J.W., Manavis J., Summersides G.E., Blumbergs P.C. (2003). Brain damage in pigs produced by impact with a nonpenetrating captive bolt pistol. Aust. Vet. J..

[8] Whiting T.L., Marion C.R. (2011). Perpetration-induced traumatic stress—A risk for veterinarians involved in the destruction of healthy animals. Can. Vet. J..

[9] Matthis J.S. (2004). Selected Employee Attributes and Perceptions Regarding Methods and Animal Welfare Concerns Associated with Swine Euthanasia. Ph.D. Thesis.

[10] McKeegan D.E.F., McIntyre J.A., Demmers T., Lowe J.C., Wathes C., van den Broek P., Coenen A.M.L., Gentle M. (2007). Physiological and behavioural responses of broilers to controlled atmosphere stunning: Implications for welfare. Anim. Welf..

[11] Rault J.-L., McMunn K.A., Marchant-Forde J.N., Lay D.C. (2013). Gas alternatives to carbon dioxide for euthanasia: A piglet perspective. J. Anim. Sci..

[12] Sadler L.J., Hagen C.D., Wang C., Widowski T.M., Johnson A.K., Millman S.T. (2014). Effects of flow rate and gas mixture on the welfare of weaned and neonate pigs during gas euthanasia. J. Anim. Sci..

[13] Velarde A., Cruz J., Gispert M., Carrion D., Ruiz de la Torre J.L., Diestre A., Manteca X. (2007). Aversion to carbon dioxide stunning in pigs: Effect of carbon dioxide concentration and halothane genotype. Anim. Welf..

[14] Hewett T.A., Kovacs M.S., Artwohl J.E., Bennett B.T. (1993). A comparison of euthanasia methods in rats, using carbon dioxide in prefilled and fixed flow-rate filled chambers. Lab. Anim. Sci..

[15] Niel L., Weary D.M. (2006). Behavioural responses of rats to gradual-fill carbon dioxide euthanasia and reduced oxygen concentrations. Appl. Anim. Behav. Sci..

[16] Pritchett K., Corrow D., Stockwell J., Smith A. (2005). Euthanasia of neonatal mice with carbon dioxide. Comp. Med..

[17] Pritchett-Corning K.R. (2009). Euthanasia of neonatal rats with carbon dioxide. J. Am. Assoc. Lab. Anim. Sci..

[18] Liotti M., Brannan S., Egan G., Shade R., Madden L., Abplanalp B., Robillard R., Lancaster J., Zamarripa F.E., Fox P.T. (2001). Brain responses associated with consciousness of breathlessness (air hunger). Proc. Natl. Acad. Sci. USA.

[19] Lansing R.W., Gracely R.H., Banzett R.B. (2009). The multiple dimensions of dyspnea: Review and hypothesis. Respir. Physiol. Neurobiol..

[20] Beausoleil N.J., Mellor D.J. (2015). Introducing breathlessness as a significant animal welfare issue. N. Z. Vet. J..

[21] Anton F., Euchner I., Handwerker H.O. (1992). Psychophysical examination of pain induced by defined CO_2_ pulses applied to the nasal mucosa. Pain.

[22] Danneman P.J., Stein S., Walshaw S.O. (1997). Humane and practical implications of using carbon dioxide mixed with oxygen for anaesthesia or euthanasia of rats. Lab. Anim. Sci..

[23] Lambooij E., Gerritzen M.A., Engal B., Hillebrand S.J.W., Lankhaar J., Pieterse C. (1999). Behavioural responses during exposure of broiler chickens to different gas mixtures. Appl. Anim. Behav. Sci..

[24] Raj A.B.M., Gregory N.G. (1996). Welfare implications of the gas stunning of pigs 2. Stress of induction of anaesthesia. Anim. Welf..

[25] Sutherland M.A., Bryer P.J., Backus B.L. (2017). The effect of age and method of gas delivery on carbon dioxide euthanasia of pigs. Anim. Welf..

[26] Freed D.L.J. (1983). CO_2_ euthanasia. Nature.

[27] Raj A.B.M., Gregory N.G. (1995). Welfare implications of the gas stunning of pigs 1. Determination of aversion to the initial inhalation of carbon dioxide or argon. Anim. Welf..

[28] Thurauf N., Friedel I., Hummel C., Kobal G. (1991). The mucosal potential elicited by noxious chemical stimuli with CO_2_ in rats: Is it a peripheral nociceptive event?. Neurosci. Lett..

[29] Peppel P., Anton F. (1993). Responses of rat medullary dorsal horn neurons following intranasal noxious chemical stimulation: Effects of stimulus intensity, duration, and interstimulus interval. J. Neurophysiol..

[30] Sadler L.J., Karriker L.A., Schwartz K.J., Johnson A.K., Widowski T.M., Wang C., Sutherland M.A., Millman S.T. (2014). Are severely depressed suckling pigs resistant to gas euthanasia?. Anim. Welf..

[31] Gibson T.J., Johnson C.B., Murrell J.C., Mitchinson S.L., Stafford K.J., Mellor D.J. (2009). Amelioration of electroencephalographic responses to slaughter by non-penetrative captive-bolt stunning after ventral-neck incision in halothane-anaesthetised calves. N. Z. Vet. J..

[32] Dalmau A., Rodriguez P., Llonch P., Velarde A. (2010). Stunning pigs with different gas mixtures: Aversion in pigs. Anim. Welf..

[33] Rodriguez P., Dalmau A., Ruiz de la Torre J.L., Manteca X., Jensen E.W., Rodriguez B.E., Litvan H., Velarde A. (2008). Assessment of unconsciousness during carbon dioxide stunning in pigs. Anim. Welf..

[34] Llonch P., Dalmau A., Rodriguez P., Manteca X., Velarde A. (2012). Aversion to nitrogen and carbon dioxide mixtures for stunning pigs. Anim. Welf..

[35] Noonan G.J., Rand J.S., Priest J., Ainscow J., Blackshaw J.K. (1994). Behavioural observations of piglets undergoing tail docking, teeth clipping and ear notching. Appl. Anim. Behav. Sci..

[36] Prunier A., Mounier A., Hay M. (2005). Effects of castration, tooth resection, or tail docking on plasma metabolites and stress hormones in young pigs. J. Anim. Sci..

[37] Carroll J.A., Berg E.L., Strauch T.A., Roberts M.P., Kattesh H.G. (2006). Hormonal profiles, behavioural responses, and short-term growth performance after castration of pigs at three, six, nine, or twelve days of age. J. Anim. Sci..

[38] Sutherland M.A., Bryer P.J., Krebs N., McGlone J.J. (2008). Tail docking in pigs: Acute physiological and behavioural responses. Animal.

[39] Meyer R.E., Whitley J.T., Morrow W.E.M., Stikeleather L.F., Baird C.L., Rice J.M., Halbert B.V., Styles D.K., Whisnant C.S. (2013). Effect of physical and inhaled euthanasia methods on hormonal measures of stress in pigs. J. Swine Health Prod..

[40] Blackmore D.K., Newhook J.C. (1982). Electroencephalographic studies of stunning and slaughter of sheep and calves‚ Part 3: The duration of insensibility induced by electrical stunning in sheep and calves. Meat Sci..

[41] Johnson C.B., Wilson P., Woodbury M., Caulkett N. (2005). Comparison of analgesic techniques for antler removal in halothane-anaesthetised red deer (Cervus elaphus): Electroencephalographic responses. Vet. Anaesth. Anal..

[42] Murrell J.C., Johnson C.B. (2006). Neurophysiological techniques to assess pain in animals. J. Vet. Pharmacol. Therapy.

[43] Gibson T.J., Johnson C.B., Stafford K.J., Mitchinson S.L., Mellor D.J. (2007). Validation of the acute electroencephalographic responses of calves to noxious stimulus with scoop dehorning. N. Z. Vet. J..

[44] Kongara K., Chambers J.P., Johnson C.B. (2010). Electroencephalographic responses of tramadol, parecoxib and morphine to acute noxious electrical stimulation in anaesthetised dogs. Res. Vet. Sci..

[45] Diesch T.J., Mellor D.J., Johnson C.B., Lentle R.G. (2009). Electroencephalographic responses to tail clamping in anaesthetized rat pups. Lab. Anim..

